# Supplementary material for: Constructing neural network models from brain data reveals representational transformations linked to adaptive behavior
Source: Nat Commun. 2022 Feb 3;13:673. doi: 10.1038/s41467-022-28323-7 (PMC8814166; doi:10.1038/s41467-022-28323-7)
Supplement: Supplementary file 1 — Supplementary Information [file 41467_2022_28323_MOESM1_ESM.pdf]

Supplementary Figures

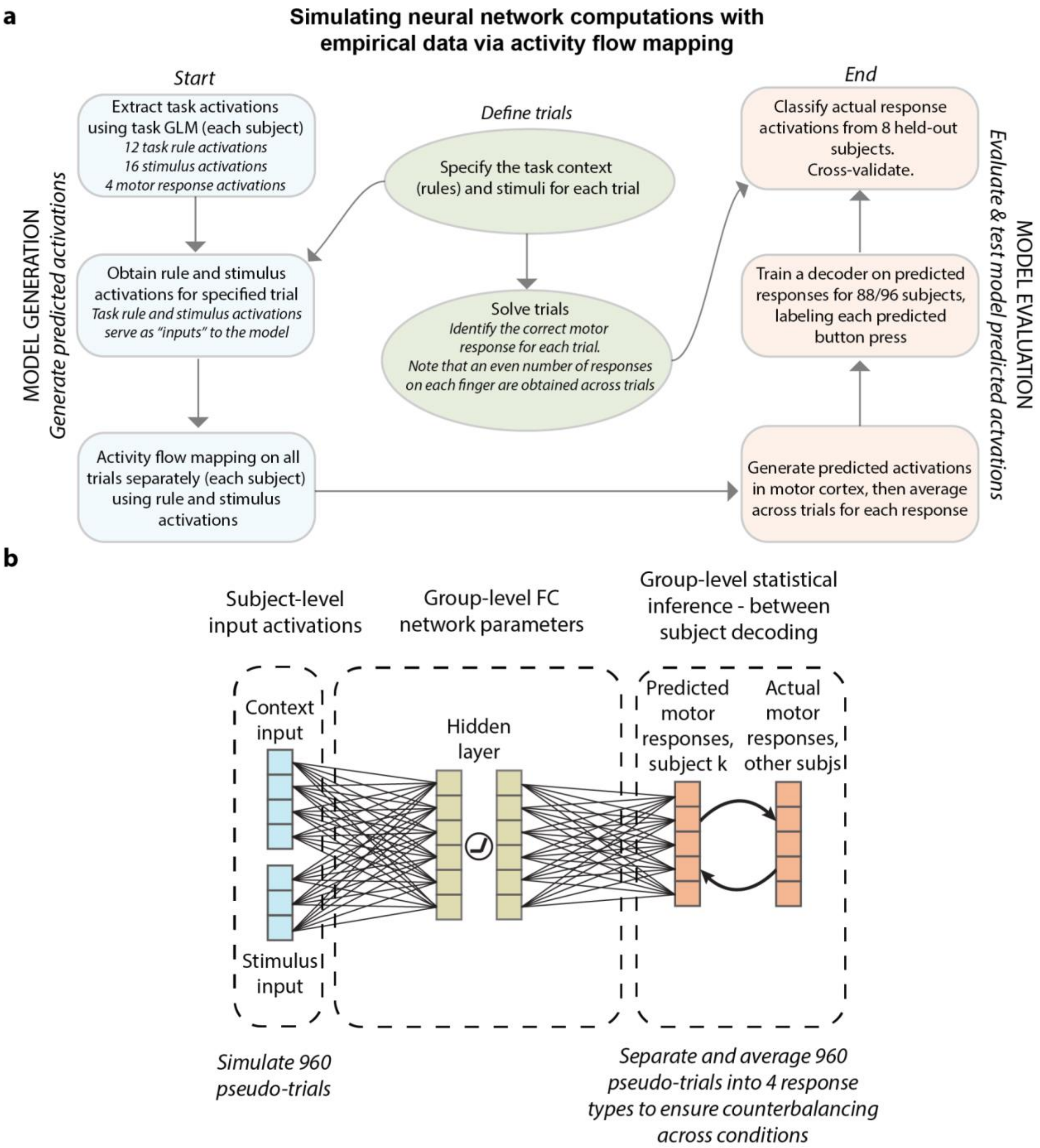

Supplementary Figure 1. Additional details describing the ENN model and simulations. a) Flow chart describing neural network simulations with empirical data via activity flow mapping**.** We generate a subject's predicted motor response activations using only task rule and sensory stimulus activation

patterns as inputs. We then test these predictions against the actual motor response activations of other subjects. **b)** Detailing subject-specific versus group-level contributions of the ENN. The ENN produced group-level inferences on transforming rule and stimulus activations into motor response activations. Group-level inferences were assessed by between-subject decoding, training a model on predicted motor responses and predicted the actual motor response activations of *other subjects* in a cross-validated fashion. The core network parameters (e.g., resting-state FC weights) were estimated at the group-level. Input activations were estimated at the subject level to ensure that group-level predictions could be performed on predictions.

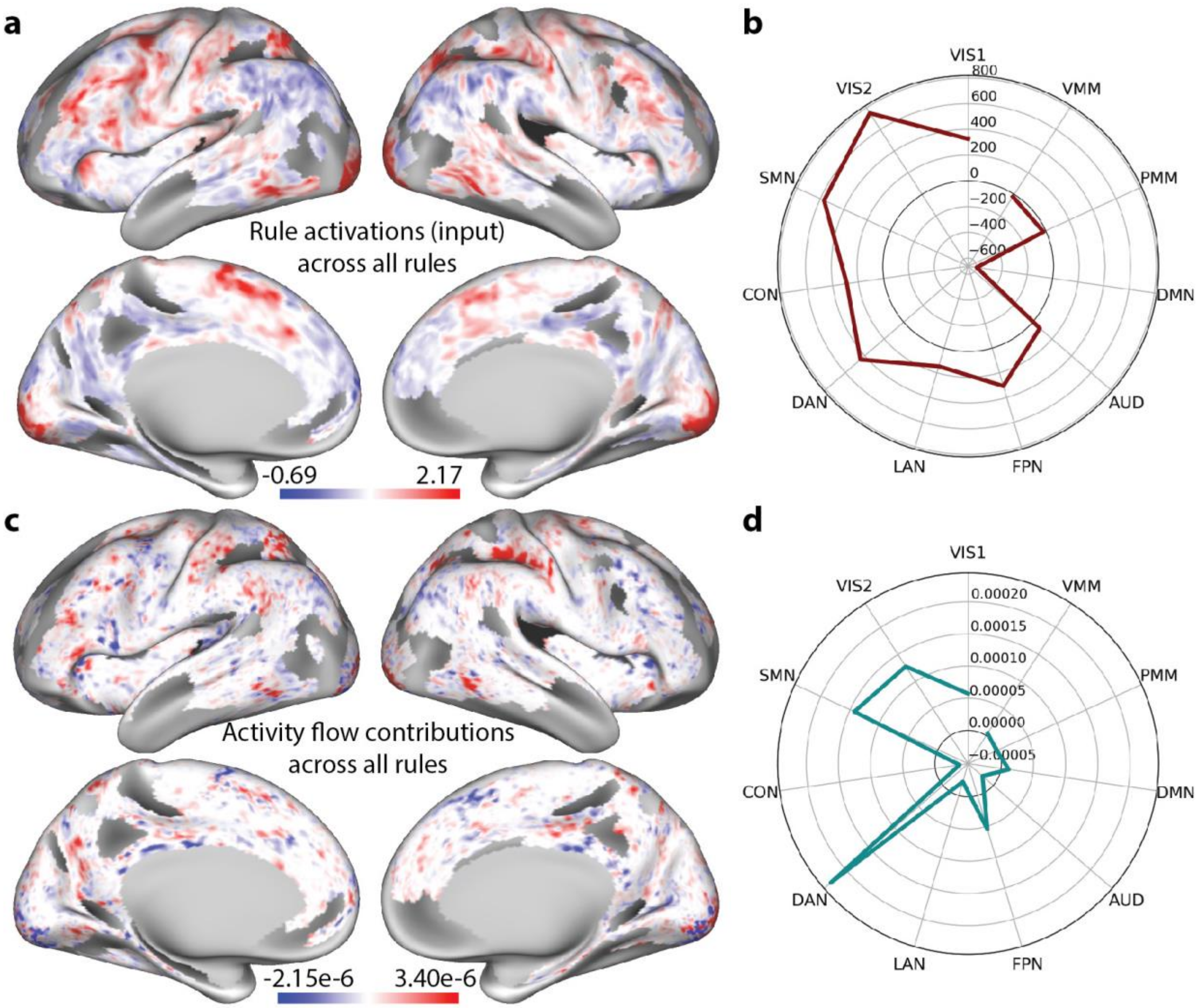

Supplementary Figure 2. Comparison of task rule activations (in task rule regions) and their activity flow contributions (activation multiplied by FC weights) onto conjunction hubs. a) The task rule activations for every vertex in the rule regions, averaged across all rule sets. This map illustrates the activations that are fed in as inputs into the ENN. Units are in task GLM beta coefficients. **b)** The distribution of task rule activations, summed across all vertices within each functional network. Both sensorimotor and association networks appear to have strong rule-related activations. **c)** The activity flow contributions (task rule activations weighted by their FC weights with conjunction hubs) of each vertex in the task rule regions. The activity flow map is noticeably different from a), given that this visualization takes into consideration both the task rule activity and their FC with conjunction hubs. This map is obtained by averaging across the activity flow contributions to all vertices in conjunction hubs. **d)** The distribution of task rule activity flow contributions to conjunction regions, summed across all vertices within each functional network. Despite having large activations in sensorimotor regions in the original task rule activation map, the activity flow contributions from these networks are dampened by their FC to conjunction hubs. The DAN contributes the most activity flow to conjunction hubs. Network abbreviations: Primary visual (VIS1), Secondary visual (VIS2), Somatomotor (SMN), Cingulo-opercular (CON), Dorsal attention (DAN), Language (LAN), Frontoparietal (FPN), Auditory (AUD), Default Mode (DMN), Posterior multi-modal (PMM), Ventral multi-modal (VMM).

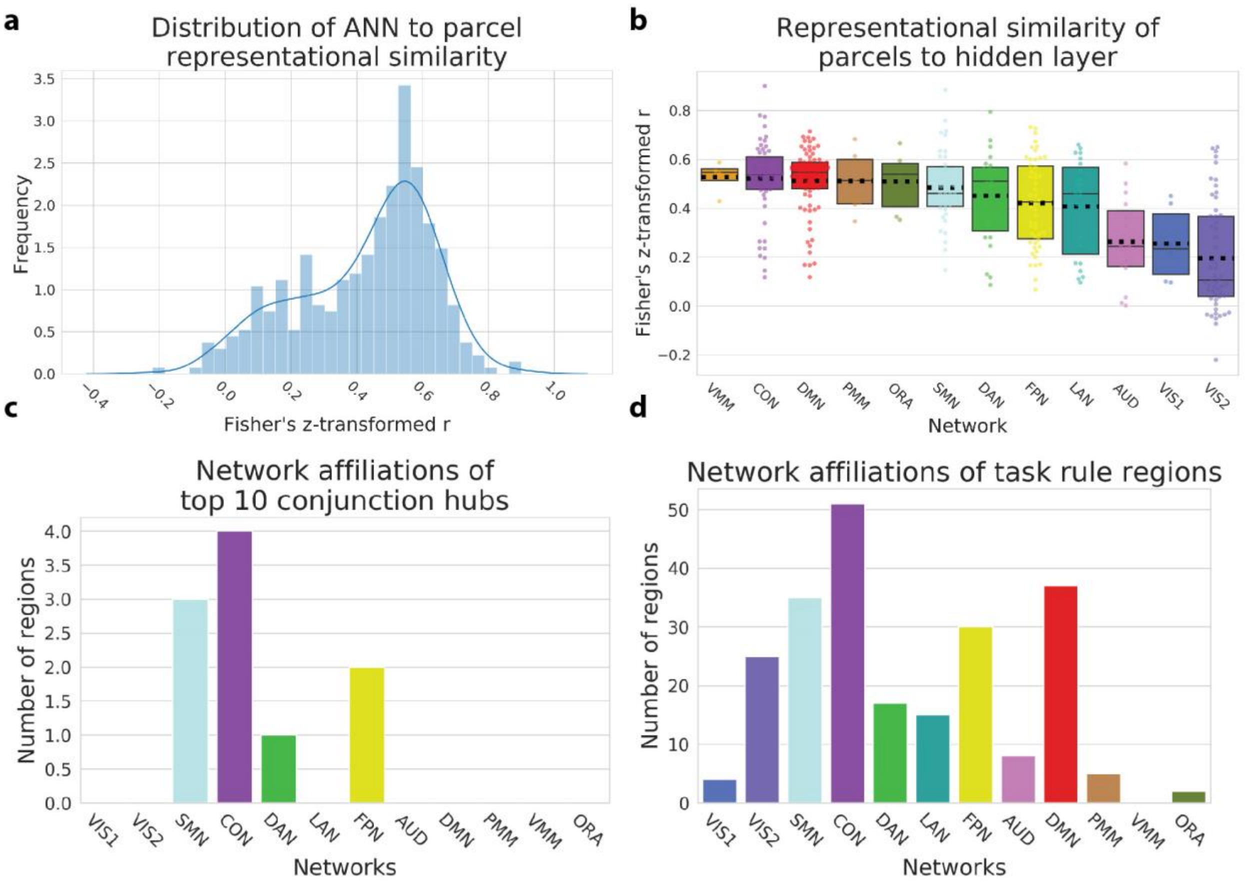

Supplementary Figure 3. Distribution and network associations of conjunction hubs and the task rule input layer using a previously defined multimodal atlas and network partition[25,26]**. a)** Histogram of the similarity (rank correlation) of the representational similarity matrix of the ANN's hidden layer and each cortical parcel (Mean of distribution=0.43). **b)** Distribution of similarity scores for each functional network (each point reflects a different parcel belonging to the functional network). Boxplots reflect the interquartiles of the distribution, black dotted line the mean, and solid line the median. Strip plots reflect the entire distribution. **c)** The network affiliations of the 10 conjunction hub brain areas. **d)** Network affiliations of the 228 brain regions that contained decodable task rule activations. Network abbreviations: Primary visual (VIS1), Secondary visual (VIS2), Somatomotor (SMN), Cingulo-opercular (CON), Dorsal attention (DAN), Language (LAN), Frontoparietal (FPN), Auditory (AUD), Default Mode (DMN), Posterior multi-modal (PMM), Ventral multi-modal (VMM), Orbito-affective (ORN). Source data are provided as a Source Data file.

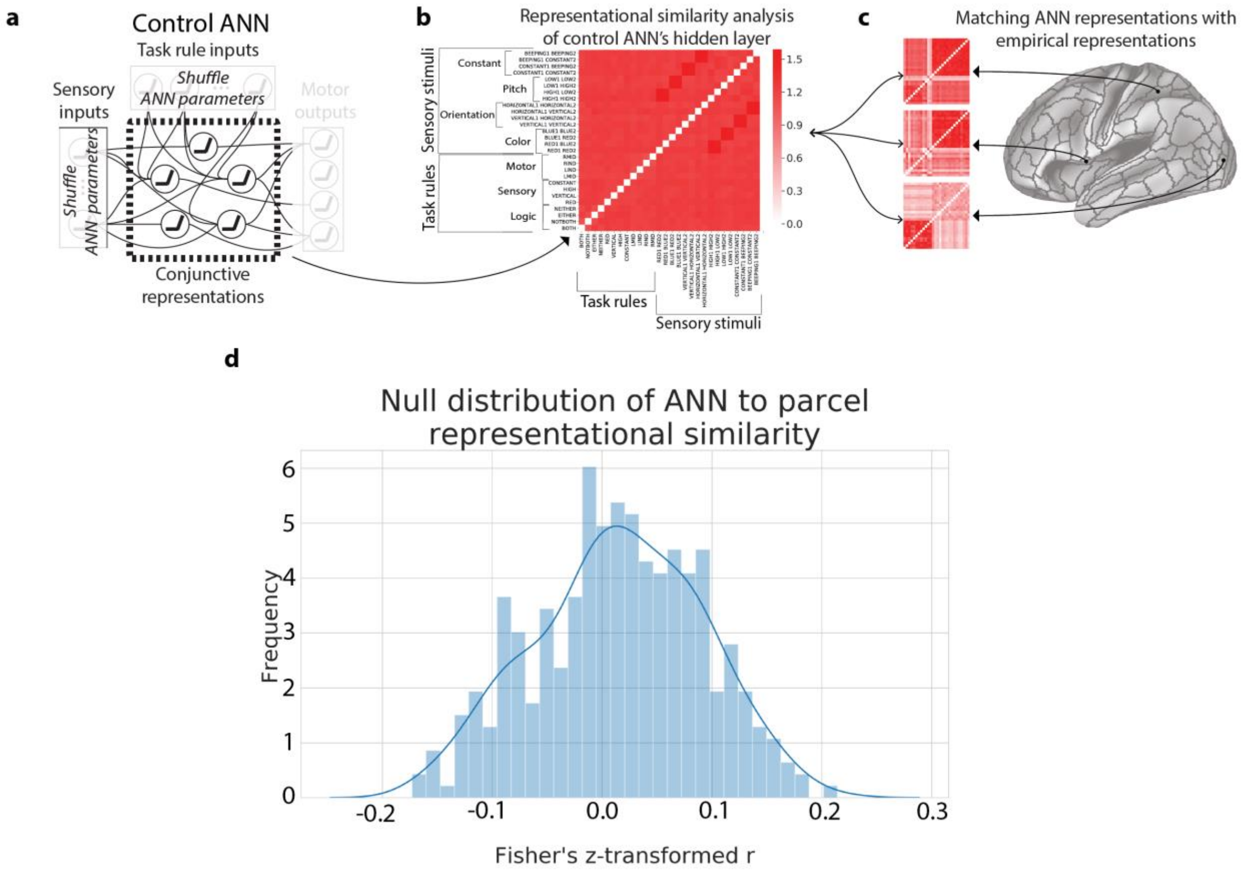

Supplementary Figure 4. Evaluating the representational similarity of each brain parcel with a control ANN with shuffled parameters (e.g., weights). a) We generated 10,000 control ANNs, where after training, we randomly shuffled the parameters (i.e., weights and biases) within each layer. This was an appropriate control since this impaired the ANN's ability to perform the task (at chance), while preserving the distribution of parameters within each layer. **b)** Using the control ANNs, we computed the averaged representational similarity matrix of the hidden layer. We found that the control ANN effectively obliterated any representational structure (dissimilarities) that were present in the unshuffled ANN (see Fig. 5b). **c)** As in Figure 5, we measured the similarity of the control ANN's RSM with the RSMs of each brain parcel. **d)** Histogram of the similarity (rank correlation) of the RSM of the control ANN's hidden layer and the RSMs of each cortical parcel (Mean of distribution=0.02). In contrast to the non-shuffled ANN's hidden layer (see Supplementary Fig. 3a), the mean similarity (across all parcels) was near 0, suggesting that the correspondence between the ANN's true hidden layer and the empirical data were highly non-trivial. Source data are provided as a Source Data file.

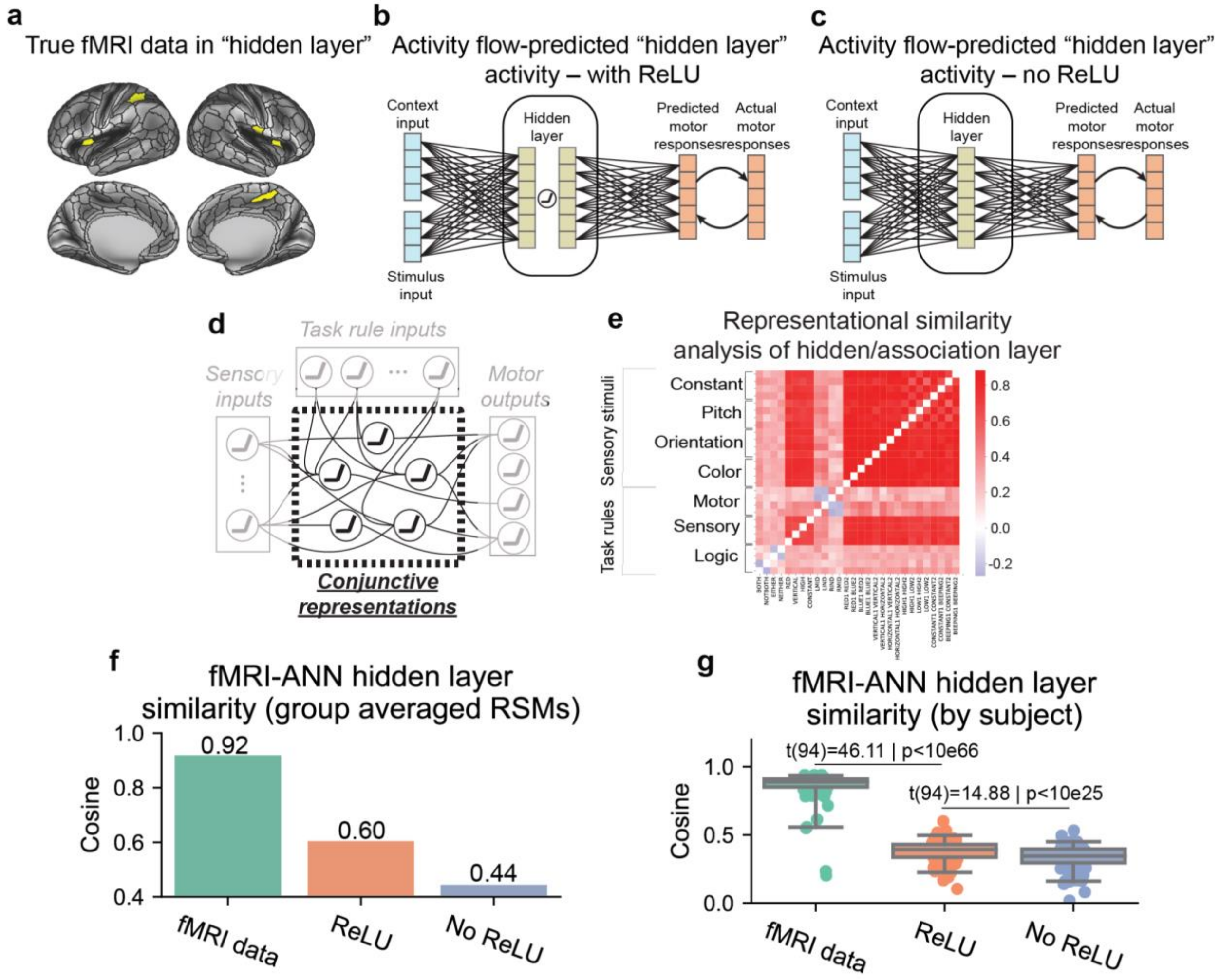

Supplementary Figure 5. Inclusion of the ReLU in the hidden layer increased the similarity of activity flow-predicted hidden representations with the conjunctive hidden representations in the ANN. We calculated the ANN's hidden layer representations with the **a)** empirical representations in the true conjunctive "hidden" layers of the fMRI data, **b)** the activity flow-predicted hidden representations in those same parcels after the ReLU was applied, and **c)** the activity flow-predicted hidden representations in those same parcels before the ReLU was applied. For the actual and predicted activations in **a-c**, we then constructed a representational similarity matrix (RSM) that contained the same exact conditions as the RSM we measured in the ANN. **d,e)** The hidden layer of the ANN with its corresponding RSM. Note that the RSM of the activity flow-predicted hidden layers was computed in the same way as the ANN – by projecting the input activations of a single condition onto the hidden layer, while fixing all other weights to 0. **f)** The similarity of the actual and activity flow-predicted RSMs with that of the ANN's RSM. Activations were averaged across subjects first, and then the RSM was constructed using the group-averaged activations for every condition. **g)** Same analysis as in **f**, but computing RSMs for each subject first, and then measuring the similarity of each subject's RSM with the ANN's RSM. The similarity of the ANN's RSM with the activity flow-predicted representations after the ReLU was always greater than the predictions without the ReLU. Boxplots maxima/minima reflect the 95% confidence interval, the box bounds define the 1st and 3rd quartiles of the distribution, and the center line indicates the median (*n*=96 subjects). Statistical testing was performed using two-sided t-tests. Source data are provided as a Source Data file.

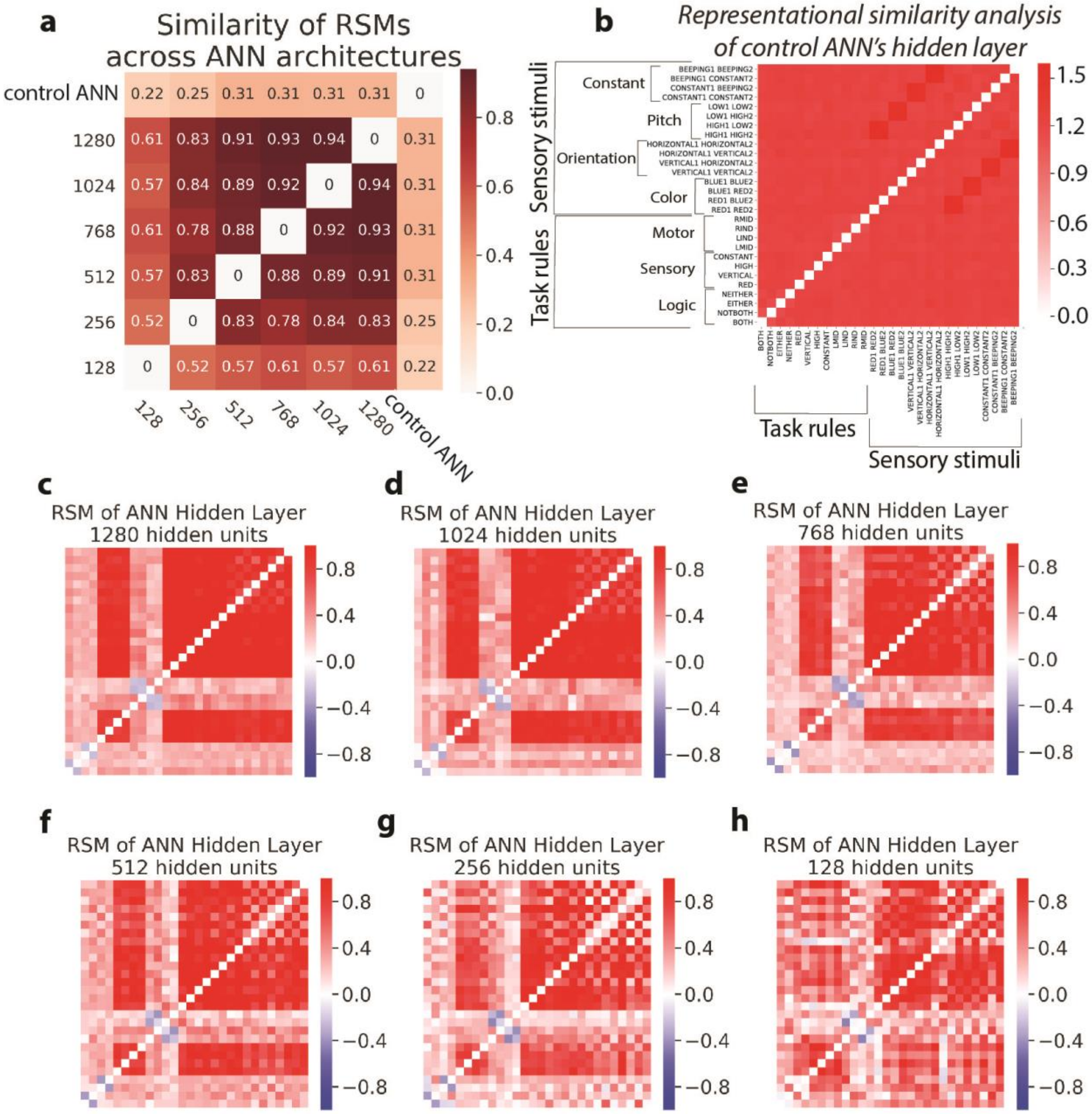

Supplementary Figure 6. Evaluating the representational similarities of the ANN's hidden layer across different ANN architectures and controls. a) The similarity of the RSMs across different ANNs with different hidden units and the control ANN (i.e., shuffled parameters). We found that ANNs with different numbers of hidden units generally had highly similar representational geometries. However, ANNs with greater hidden units tended to have greater similarity, likely due to improved generalization[68]. **b)** The RSM of the control ANN (shuffled parameters within each layer). **c-h)** The RSMs of ANNs with 1280, 1024, 768, 512, 256, and 128 hidden units. We trained a single instance of each model (trained until accuracy>99.5%) prior to visualizing its RSMs. RSMs with fewer hidden units tended to have more variable (noisier) RSMs across initializations.

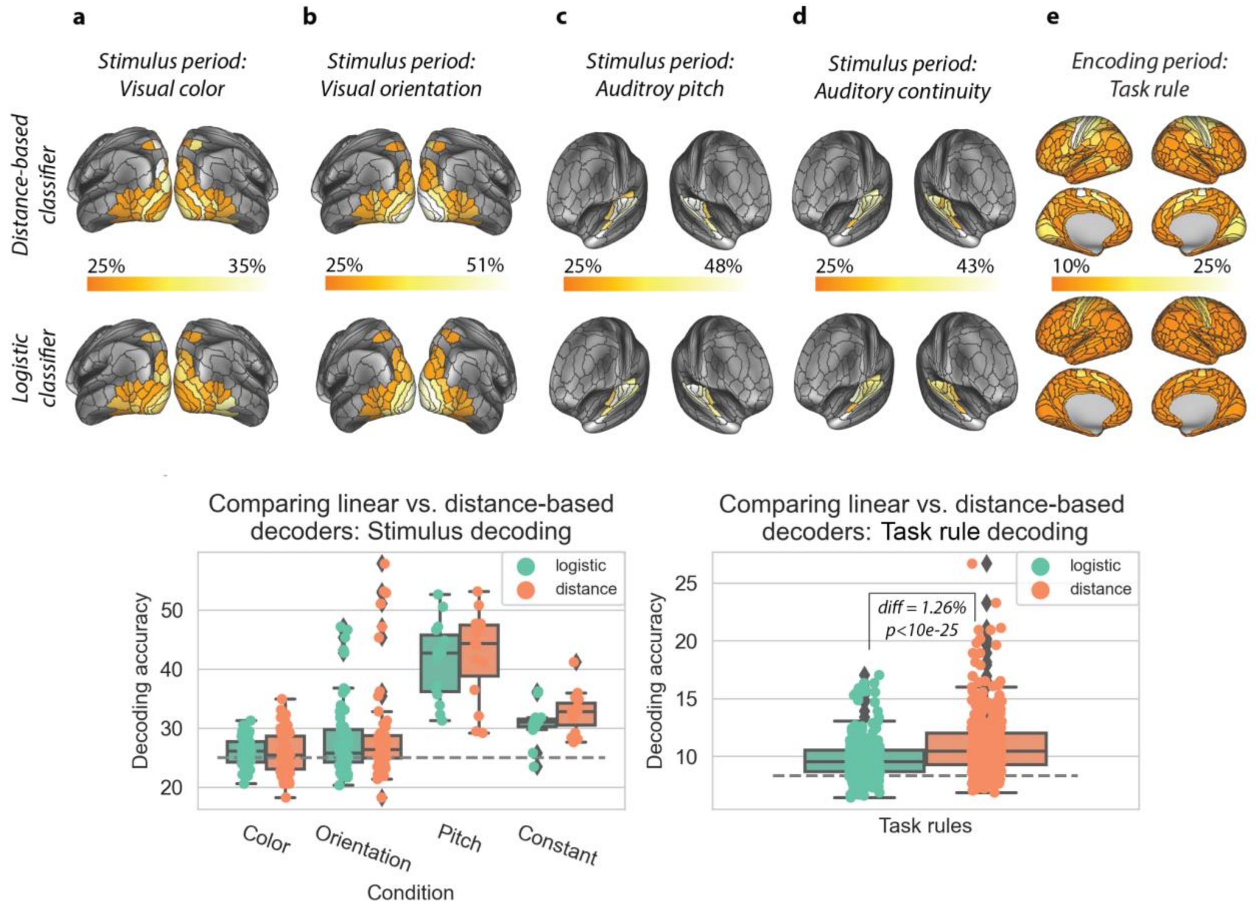

Supplementary Figure 7. Comparing a linear versus distance-based decoder for identifying regions containing stimulus and task rule features. a-e) Surface visualizations for decoding accuracies of each parcel (using vertices within each parcel) for **a)** color (visual), **b)** orientation (visual), **c)** pitch (auditory), **d)** continuity/constant (auditory), and **e)** task rule (12-way decoding). Top surface plots employ the distance-based classifier. Bottom plots employ a logistic classifier. **f)** Comparing overall decoding accuracies for all regions of interests for stimulus features (n_{color}=60, $n_{orientation}$=60, n_{pitch}=15, $n_{constant}$=15). Plot includes unthresholded classification accuracies. Since stimulus decoding involved a 4-way classification, chance was 25%. Overall, there were no statistically different decoding accuracies for distance-based versus linear logistic decoders. **g)** For task rule classifications, distance-based decoders outperformed logistic classifiers (average accuracy difference +1.26%, p<10e-25 using a two-sided Wilcoxon signed-rank test). Overall, distance-based decoders perform similarly or better than logistic decoders for our data set (*n*=360). (Distance-based decoders were also computationally cheaper.) Boxplots maxima/minima reflect the 95% confidence interval, the box bounds define the 1st and 3rd quartiles of the distribution, and the center line indicates the median (*n*=96 subjects). Source data are provided as a Source Data file.

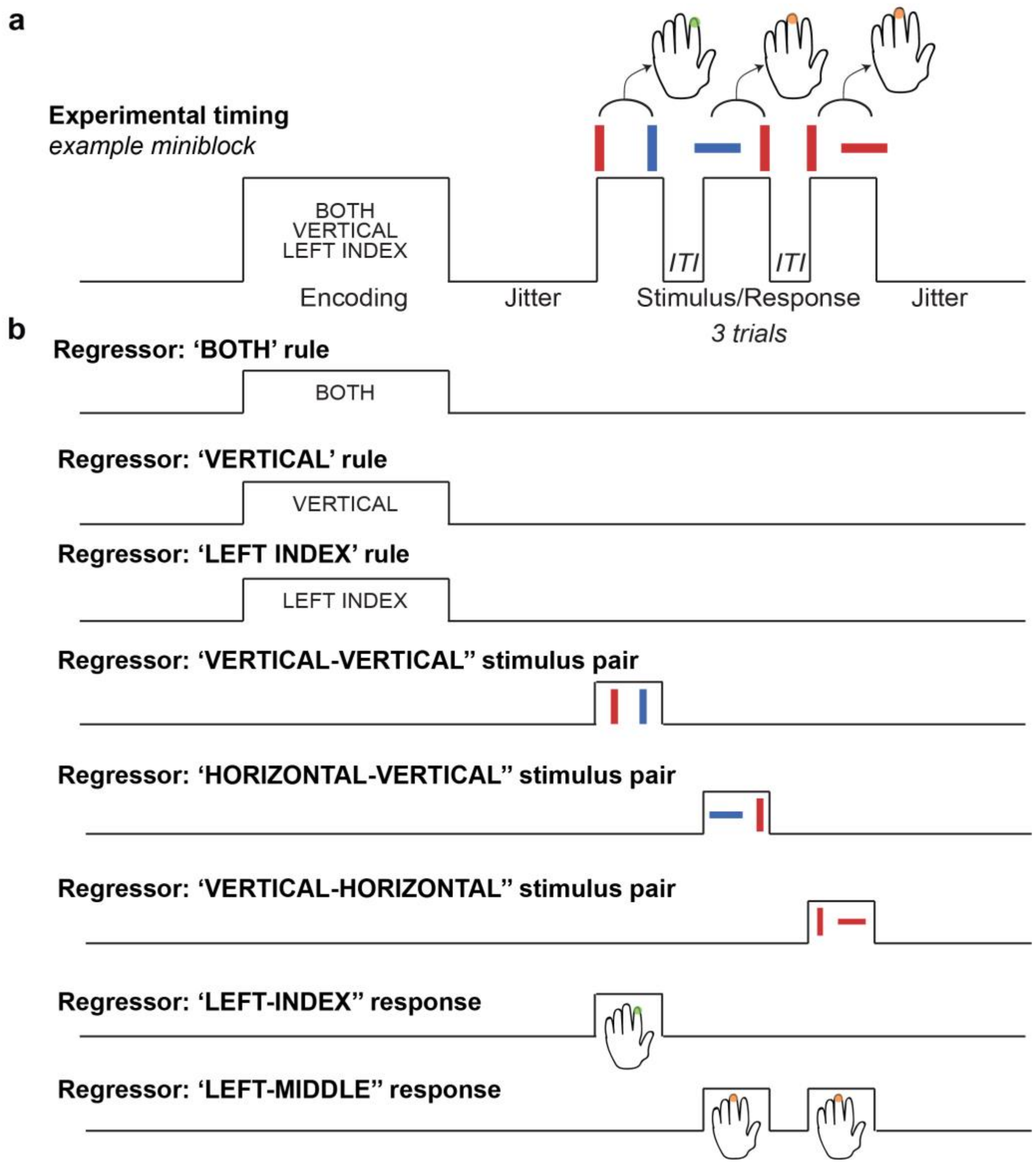

Supplementary Figure 8. Example of task GLM approach to obtain task activation estimates. a) An example miniblock containing one encoding block (task rule set) and three trials. Note that while stimulus presentation and response periods overlap, they are properly counterbalanced across trials. **b)** The regressors for the relevant task conditions in the example miniblock. We obtain regressors (estimated across all 128 miniblocks) for all task rule, sensory stimuli, and motor response conditions. Altogether there are 32 different task conditions (12 task rules, 16 sensory stimuli pairs, and four motor response periods). Note that task rule regressors (logic, sensory, and motor rule examples) appear collinear in this example, but that across all 128 miniblocks task rule conditions are properly counterbalanced to avoid biasing towards any specific rule combination. Regressors shown here are illustrated without convolution with SPM's canonical HRF.

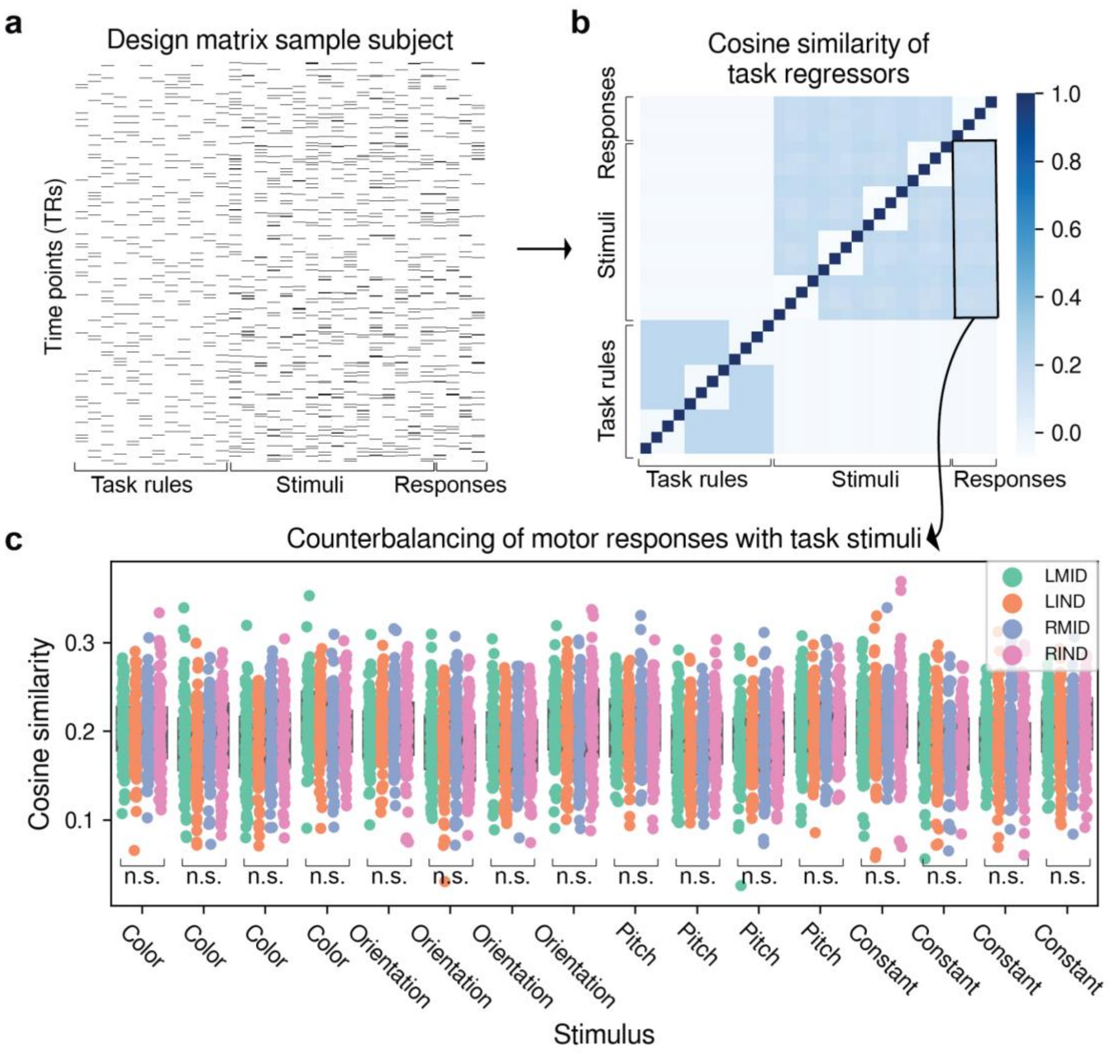

Supplementary Figure 9. Counterbalancing (and averaging) across conditions ensures statistical independence of activation measures between stimulus and response conditions. a) A task GLM design matrix for an example subject. For task GLMs, all conditions (rules, stimuli, responses) were modeled simultaneously using multiple linear regression, increasing the statistical independence of estimates (in addition to counterbalancing). **b)** Group-averaged cosine similarity matrix of task regressors. For each subject, we estimated the cosine similarity of task regressors, then averaged across subjects. Naturally, the diagonal has a cosine similarity of 1. There is no interdependence between task rules and stimulus/motor responses, since the relevant task timing intervals do not overlap. However, for motor and stimulus conditions, there are some weak dependencies (by necessity), since the response intervals overlap with stimulus presentations. However, due to the counterbalancing of stimulus and response types, regressors/conditions will remain independent when modeled together in a task GLM. Therefore, simultaneous modeling of all stimulus and response conditions in a task GLM will ensure unique variance is assigned to each condition. This is because multiple linear regression conditions on all other regressors/conditions. **c)** No main effect of stimulus-response similarity across subjects, demonstrating that averaging across these counterbalanced stimuli eliminates any biases toward particular motor responses. For every stimulus-response pairing, we performed a one-way ANOVA to test the null hypothesis that across response conditions, the distributions had different means (subjects as a random

effect). For every stimulus condition, we could not reject the null, indicating no bias towards any given stimulus-response pairing (all $p>0.05$, FDR-corrected).

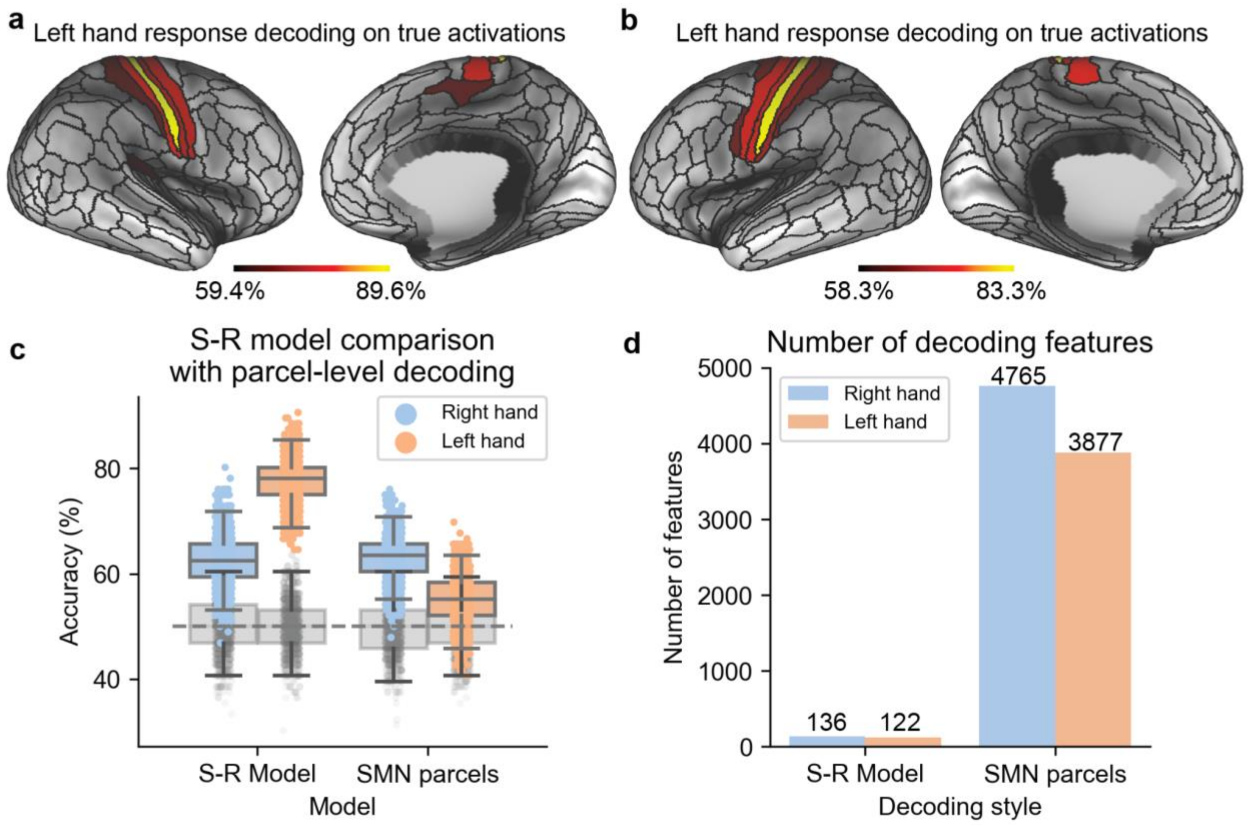

e

	Accuracy (RH)	P-value (RH)	Accuracy (LH)	P-value (LH)
**S-R Model**	62.65	0.029667	77.58	0.000018
**SMN parcels**	62.98	0.022551	54.92	0.211641

Supplementary Figure 10. Using classification of response activations to identify motor output parcels reduces spatial specificity of digit representations and reduces stimulus-response decodability for left hand responses. a) Left and **b)** right hand response decoding accuracy on the true fMRI response activations. Classifications were limited to the somatomotor network (SMN) on the appropriate hemisphere (e.g., right hemisphere for left hand responses). **c)** S-R model decoding accuracy on the output parcels using the full ENN model (on the left, as presented in the main manuscript; Fig 7b). On the right, the classification accuracy using the SMN parcels identified in **a** and **b**. Right hand classification accuracies remain the same, while left-hand accuracies are degraded. All distributions *n*=1000. Boxplots maxima/minima reflect the 95% confidence interval, the box bounds define the 1st and 3rd quartiles of the distribution, and the center line indicates the median. **d)** Number of decoding features that feed the decoder on the output layer. Inclusion of all SMN parcels significantly increases the number of features the decoder is required to distinguish. **e)** Table of classification accuracies for each model. Cross-validation details are exactly the same as reported in Fig. 7. Statistical testing was performed with one-sided permutation testing (*n*=1000 label permutations). Source data are provided as a Source Data file.

Supplementary Table 1. Regions containing decodable color (red/blue) stimulus activations. Bold text indicates column headers.

**Label**	**GlasserID**	**Network Affiliation**	**Hemisphere**
Visual2-54_L-Ctx	L_VVC	Visual2	L
Visual2-05_R-Ctx	R_V4	Visual2	R
Visual1-03_R-Ctx	R_DVT	Visual1	R
Visual2-22_R-Ctx	R_V4t	Visual2	R

Supplementary Table 2. Regions containing decodable orientation (vertical/horizontal) stimulus activations. Bold text indicates column headers.

**Label**	**GlasserID**	**Network Affiliation**	**Hemisphere**
Visual1-04_L-Ctx	L_V1	Visual1	L
Visual2-28_L-Ctx	L_MST	Visual2	L
Visual2-30_L-Ctx	L_V2	Visual2	L
Visual2-31_L-Ctx	L_V3	Visual2	L
Visual2-32_L-Ctx	L_V4	Visual2	L
Visual2-33_L-Ctx	L_V8	Visual2	L
Visual2-35_L-Ctx	L_V7	Visual2	L
Visual2-40_L-Ctx	L_LO2	Visual2	L
Visual2-41_L-Ctx	L_PIT	Visual2	L
Visual2-42_L-Ctx	L_MT	Visual2	L
Visual2-51_L-Ctx	L_V3CD	Visual2	L
Visual1-01_R-Ctx	R_V1	Visual1	R
Visual2-03_R-Ctx	R_V2	Visual2	R
Visual2-04_R-Ctx	R_V3	Visual2	R
Visual2-05_R-Ctx	R_V4	Visual2	R
Visual2-06_R-Ctx	R_V8	Visual2	R
Visual2-09_R-Ctx	R_IPS1	Visual2	R
Visual2-12_R-Ctx	R_LO1	Visual2	R

Supplementary Table 3. Regions containing decodable pitch (high/low) stimulus activations. Bold text indicates column headers.

**Label**	**GlasserID**	**Network Affiliation**	**Hemisphere**
Auditory-08_L-Ctx	L_A1	Auditory	L
Auditory-09_L-Ctx	L_52	Auditory	L
Auditory-10_L-Ctx	L_RI	Auditory	L
Auditory-11_L-Ctx	L_TA2	Auditory	L
Auditory-12_L-Ctx	L_PBelt	Auditory	L
Auditory-13_L-Ctx	L_MBelt	Auditory	L
Auditory-14_L-Ctx	L_LBelt	Auditory	L
Auditory-15_L-Ctx	L_A4	Auditory	L
Auditory-01_R-Ctx	R_A1	Auditory	R
Auditory-02_R-Ctx	R_52	Auditory	R
Auditory-03_R-Ctx	R_TA2	Auditory	R
Auditory-04_R-Ctx	R_PBelt	Auditory	R
Auditory-05_R-Ctx	R_MBelt	Auditory	R
Auditory-06_R-Ctx	R_LBelt	Auditory	R
Auditory-07_R-Ctx	R_A4	Auditory	R

Supplementary Table 4. Regions containing decodable constant/beeping stimulus activations. Bold text indicates column headers.

**Label**	**GlasserID**	**Network Affiliation**	**Hemisphere**
Auditory-08_L-Ctx	L_A1	Auditory	L
Auditory-09_L-Ctx	L_52	Auditory	L
Auditory-10_L-Ctx	L_RI	Auditory	L
Auditory-11_L-Ctx	L_TA2	Auditory	L
Auditory-12_L-Ctx	L_PBelt	Auditory	L
Auditory-13_L-Ctx	L_MBelt	Auditory	L
Auditory-14_L-Ctx	L_LBelt	Auditory	L
Auditory-15_L-Ctx	L_A4	Auditory	L
Auditory-01_R-Ctx	R_A1	Auditory	R
Auditory-02_R-Ctx	R_52	Auditory	R
Auditory-03_R-Ctx	R_TA2	Auditory	R
Auditory-04_R-Ctx	R_PBelt	Auditory	R
Auditory-05_R-Ctx	R_MBelt	Auditory	R
Auditory-06_R-Ctx	R_LBelt	Auditory	R
Auditory-07_R-Ctx	R_A4	Auditory	R

Supplementary Table 5. Conjunction hubs. Bold text indicates column headers.

**Label**	**GlasserID**	**Network Affiliation**	**Hemisphere**
Cingulo-Opercular-49_L-Ctx	L_FOP3	Cingulo-Opercular	L
Somatomotor-15_R-Ctx	R_OP4	Somatomotor	R
Dorsal-Attention-18_L-Ctx	L_AIP	Dorsal-Attention	L
Cingulo-Opercular-04_R-Ctx	R_5mv	Cingulo-Opercular	R
Cingulo-Opercular-22_R-Ctx	R_FOP3	Cingulo-Opercular	R
Somatomotor-08_R-Ctx	R_7PC	Somatomotor	R
Cingulo-Opercular-44_L-Ctx	L_PFcm	Cingulo-Opercular	L
Frontoparietal-34_L-Ctx	L_a47r	Frontoparietal	L
Frontoparietal-03_R-Ctx	R_7Pm	Frontoparietal	R
Somatomotor-07_R-Ctx	R_7AL	Somatomotor	R

Supplementary Table 6. Task rule (input) regions. Bold text indicates column headers.

**Label**	**GlasserID**	**Network Affiliation**	**Hemisphere**
Visual1-04_L-Ctx	L_V1	Visual1	L
Visual2-30_L-Ctx	L_V2	Visual2	L
Visual2-31_L-Ctx	L_V3	Visual2	L
Visual2-32_L-Ctx	L_V4	Visual2	L
Somatomotor-21_L-Ctx	L_4	Somatomotor	L
Somatomotor-22_L-Ctx	L_3b	Somatomotor	L
Cingulo-Opercular-30_L-Ctx	L_FEF	Cingulo-Opercular	L
Dorsal-Attention-12_L-Ctx	L_PEF	Dorsal-Attention	L
Language-10_L-Ctx	L_55b	Language	L
Frontoparietal-29_L-Ctx	L_RSC	Frontoparietal	L
Frontoparietal-30_L-Ctx	L_POS2	Frontoparietal	L
Visual2-35_L-Ctx	L_V7	Visual2	L
Visual2-36_L-Ctx	L_IPS1	Visual2	L
Visual2-38_L-Ctx	L_V3B	Visual2	L
Visual2-42_L-Ctx	L_MT	Visual2	L
Auditory-08_L-Ctx	L_A1	Auditory	L
Language-11_L-Ctx	L_PSL	Language	L
Language-12_L-Ctx	L_SFL	Language	L
Posterior-Multimodal-05_L-Ctx	L_PCV	Posterior-Multimodal	L
Default-38_L-Ctx	L_7m	Default	L
Default-39_L-Ctx	L_POS1	Default	L
Default-40_L-Ctx	L_23d	Default	L
Default-41_L-Ctx	L_v23ab	Default	L
Default-42_L-Ctx	L_d23ab	Default	L
Cingulo-Opercular-32_L-Ctx	L_23c	Cingulo-Opercular	L
Somatomotor-25_L-Ctx	L_24dd	Somatomotor	L
Somatomotor-26_L-Ctx	L_24dv	Somatomotor	L
Somatomotor-27_L-Ctx	L_7AL	Somatomotor	L
Cingulo-Opercular-33_L-Ctx	L_SCEF	Cingulo-Opercular	L
Cingulo-Opercular-34_L-Ctx	L_6ma	Cingulo-Opercular	L
Cingulo-Opercular-35_L-Ctx	L_7Am	Cingulo-Opercular	L
Somatomotor-28_L-Ctx	L_7PC	Somatomotor	L
Visual2-43_L-Ctx	L_LIPv	Visual2	L
Visual2-44_L-Ctx	L_VIP	Visual2	L
Somatomotor-29_L-Ctx	L_1	Somatomotor	L
Somatomotor-30_L-Ctx	L_2	Somatomotor	L
Somatomotor-31_L-Ctx	L_3a	Somatomotor	L
Somatomotor-32_L-Ctx	L_6d	Somatomotor	L
Somatomotor-33_L-Ctx	L_6mp	Somatomotor	L
Somatomotor-34_L-Ctx	L_6v	Somatomotor	L
Cingulo-Opercular-36_L-Ctx	L_p24pr	Cingulo-Opercular	L
Cingulo-Opercular-37_L-Ctx	L_33pr	Cingulo-Opercular	L
Cingulo-Opercular-38_L-Ctx	L_a24pr	Cingulo-Opercular	L

Cingulo-Opercular-39_L-Ctx	L_p32pr	Cingulo-Opercular	L
Default-44_L-Ctx	L_a24	Default	L
Default-45_L-Ctx	L_d32	Default	L
Frontoparietal-32_L-Ctx	L_8BM	Frontoparietal	L
Default-47_L-Ctx	L_10r	Default	L
Default-49_L-Ctx	L_8Av	Default	L
Default-51_L-Ctx	L_9m	Default	L
Default-52_L-Ctx	L_8BL	Default	L
Default-54_L-Ctx	L_10d	Default	L
Frontoparietal-33_L-Ctx	L_8C	Frontoparietal	L
Language-14_L-Ctx	L_44	Language	L
Language-15_L-Ctx	L_45	Language	L
Default-55_L-Ctx	L_47l	Default	L
Frontoparietal-34_L-Ctx	L_a47r	Frontoparietal	L
Cingulo-Opercular-40_L-Ctx	L_6r	Cingulo-Opercular	L
Language-16_L-Ctx	L_IFJa	Language	L
Frontoparietal-35_L-Ctx	L_IFJp	Frontoparietal	L
Language-17_L-Ctx	L_IFSp	Language	L
Frontoparietal-36_L-Ctx	L_IFSa	Frontoparietal	L
Frontoparietal-37_L-Ctx	L_p9-46v	Frontoparietal	L
Cingulo-Opercular-42_L-Ctx	L_9-46d	Cingulo-Opercular	L
Default-56_L-Ctx	L_9a	Default	L
Frontoparietal-39_L-Ctx	L_a10p	Frontoparietal	L
Frontoparietal-40_L-Ctx	L_11l	Frontoparietal	L
Dorsal-Attention-15_L-Ctx	L_LIPd	Dorsal-Attention	L
Dorsal-Attention-16_L-Ctx	L_6a	Dorsal-Attention	L
Frontoparietal-42_L-Ctx	L_i6-8	Frontoparietal	L
Cingulo-Opercular-43_L-Ctx	L_43	Cingulo-Opercular	L
Somatomotor-35_L-Ctx	L_OP4	Somatomotor	L
Somatomotor-36_L-Ctx	L_OP1	Somatomotor	L
Somatomotor-37_L-Ctx	L_OP2-3	Somatomotor	L
Auditory-09_L-Ctx	L_52	Auditory	L
Auditory-10_L-Ctx	L_RI	Auditory	L
Cingulo-Opercular-44_L-Ctx	L_PFcm	Cingulo-Opercular	L
Cingulo-Opercular-45_L-Ctx	L_PoI2	Cingulo-Opercular	L
Auditory-11_L-Ctx	L_TA2	Auditory	L
Cingulo-Opercular-46_L-Ctx	L_FOP4	Cingulo-Opercular	L
Cingulo-Opercular-47_L-Ctx	L_MI	Cingulo-Opercular	L
Frontoparietal-44_L-Ctx	L_AVI	Frontoparietal	L
Orbito-Affective-05_L-Ctx	L_AAIC	Orbito-Affective	L
Cingulo-Opercular-48_L-Ctx	L_FOP1	Cingulo-Opercular	L
Cingulo-Opercular-49_L-Ctx	L_FOP3	Cingulo-Opercular	L
Somatomotor-38_L-Ctx	L_FOP2	Somatomotor	L
Dorsal-Attention-17_L-Ctx	L_PFt	Dorsal-Attention	L
Dorsal-Attention-18_L-Ctx	L_AIP	Dorsal-Attention	L
Default-62_L-Ctx	L_PreS	Default	L

Language-18_L-Ctx	L_STGa	Language	L
Language-19_L-Ctx	L_A5	Language	L
Dorsal-Attention-19_L-Ctx	L_PHA3	Dorsal-Attention	L
Language-21_L-Ctx	L_STSdp	Language	L
Default-65_L-Ctx	L_STSvp	Default	L
Frontoparietal-45_L-Ctx	L_TE1p	Frontoparietal	L
Dorsal-Attention-20_L-Ctx	L_TE2p	Dorsal-Attention	L
Dorsal-Attention-21_L-Ctx	L_PHT	Dorsal-Attention	L
Visual2-45_L-Ctx	L_PH	Visual2	L
Language-22_L-Ctx	L_TPOJ1	Language	L
Posterior-Multimodal-06_L-Ctx	L_TPOJ2	Posterior-Multimodal	L
Visual1-06_L-Ctx	L_DVT	Visual1	L
Dorsal-Attention-22_L-Ctx	L_PGp	Dorsal-Attention	L
Frontoparietal-47_L-Ctx	L_IP1	Frontoparietal	L
Dorsal-Attention-23_L-Ctx	L_IP0	Dorsal-Attention	L
Cingulo-Opercular-50_L-Ctx	L_PFop	Cingulo-Opercular	L
Cingulo-Opercular-51_L-Ctx	L_PF	Cingulo-Opercular	L
Frontoparietal-48_L-Ctx	L_PFm	Frontoparietal	L
Default-69_L-Ctx	L_PGi	Default	L
Default-70_L-Ctx	L_PGs	Default	L
Visual2-46_L-Ctx	L_V6A	Visual2	L
Default-71_L-Ctx	L_PHA2	Default	L
Default-73_L-Ctx	L_31a	Default	L
Visual2-54_L-Ctx	L_VVC	Visual2	L
Cingulo-Opercular-52_L-Ctx	L_PoI1	Cingulo-Opercular	L
Somatomotor-39_L-Ctx	L_Ig	Somatomotor	L
Cingulo-Opercular-53_L-Ctx	L_FOP5	Cingulo-Opercular	L
Frontoparietal-50_L-Ctx	L_p47r	Frontoparietal	L
Auditory-14_L-Ctx	L_LBelt	Auditory	L
Auditory-15_L-Ctx	L_A4	Auditory	L
Default-77_L-Ctx	L_TE1m	Default	L
Cingulo-Opercular-55_L-Ctx	L_a32pr	Cingulo-Opercular	L
Cingulo-Opercular-56_L-Ctx	L_p24	Cingulo-Opercular	L
Visual1-01_R-Ctx	R_V1	Visual1	R
Visual2-02_R-Ctx	R_V6	Visual2	R
Visual2-03_R-Ctx	R_V2	Visual2	R
Visual2-04_R-Ctx	R_V3	Visual2	R
Visual2-05_R-Ctx	R_V4	Visual2	R
Somatomotor-01_R-Ctx	R_4	Somatomotor	R
Somatomotor-02_R-Ctx	R_3b	Somatomotor	R
Cingulo-Opercular-01_R-Ctx	R_FEF	Cingulo-Opercular	R
Cingulo-Opercular-02_R-Ctx	R_PEF	Cingulo-Opercular	R
Frontoparietal-01_R-Ctx	R_RSC	Frontoparietal	R
Frontoparietal-02_R-Ctx	R_POS2	Frontoparietal	R
Visual2-08_R-Ctx	R_V7	Visual2	R
Visual2-09_R-Ctx	R_IPS1	Visual2	R

Visual2-11_R-Ctx	R_V3B	Visual2	R
Visual2-12_R-Ctx	R_LO1	Visual2	R
Cingulo-Opercular-03_R-Ctx	R_PSL	Cingulo-Opercular	R
Posterior-Multimodal-01_R-Ctx	R_PCV	Posterior-Multimodal	R
Default-01_R-Ctx	R_7m	Default	R
Default-02_R-Ctx	R_POS1	Default	R
Default-03_R-Ctx	R_23d	Default	R
Default-04_R-Ctx	R_v23ab	Default	R
Default-05_R-Ctx	R_d23ab	Default	R
Somatomotor-03_R-Ctx	R_5m	Somatomotor	R
Cingulo-Opercular-04_R-Ctx	R_5mv	Cingulo-Opercular	R
Cingulo-Opercular-05_R-Ctx	R_23c	Cingulo-Opercular	R
Somatomotor-04_R-Ctx	R_5L	Somatomotor	R
Somatomotor-05_R-Ctx	R_24dd	Somatomotor	R
Somatomotor-06_R-Ctx	R_24dv	Somatomotor	R
Somatomotor-07_R-Ctx	R_7AL	Somatomotor	R
Cingulo-Opercular-06_R-Ctx	R_SCEF	Cingulo-Opercular	R
Cingulo-Opercular-07_R-Ctx	R_6ma	Cingulo-Opercular	R
Cingulo-Opercular-08_R-Ctx	R_7Am	Cingulo-Opercular	R
Somatomotor-08_R-Ctx	R_7PC	Somatomotor	R
Visual2-16_R-Ctx	R_LIPv	Visual2	R
Visual2-17_R-Ctx	R_VIP	Visual2	R
Dorsal-Attention-02_R-Ctx	R_MIP	Dorsal-Attention	R
Somatomotor-09_R-Ctx	R_1	Somatomotor	R
Somatomotor-10_R-Ctx	R_2	Somatomotor	R
Somatomotor-11_R-Ctx	R_3a	Somatomotor	R
Somatomotor-12_R-Ctx	R_6d	Somatomotor	R
Somatomotor-13_R-Ctx	R_6mp	Somatomotor	R
Somatomotor-14_R-Ctx	R_6v	Somatomotor	R
Cingulo-Opercular-09_R-Ctx	R_p24pr	Cingulo-Opercular	R
Cingulo-Opercular-10_R-Ctx	R_a24pr	Cingulo-Opercular	R
Cingulo-Opercular-11_R-Ctx	R_p32pr	Cingulo-Opercular	R
Frontoparietal-05_R-Ctx	R_d32	Frontoparietal	R
Frontoparietal-06_R-Ctx	R_8BM	Frontoparietal	R
Default-11_R-Ctx	R_8Av	Default	R
Default-12_R-Ctx	R_8Ad	Default	R
Default-13_R-Ctx	R_9m	Default	R
Default-14_R-Ctx	R_8BL	Default	R
Frontoparietal-07_R-Ctx	R_8C	Frontoparietal	R
Frontoparietal-08_R-Ctx	R_44	Frontoparietal	R
Default-17_R-Ctx	R_47l	Default	R
Cingulo-Opercular-12_R-Ctx	R_6r	Cingulo-Opercular	R
Language-04_R-Ctx	R_IFJa	Language	R
Frontoparietal-11_R-Ctx	R_IFSp	Frontoparietal	R
Cingulo-Opercular-13_R-Ctx	R_IFSa	Cingulo-Opercular	R
Frontoparietal-12_R-Ctx	R_p9-46v	Frontoparietal	R

Cingulo-Opercular-15_R-Ctx	R_9-46d	Cingulo-Opercular	R
Dorsal-Attention-04_R-Ctx	R_6a	Dorsal-Attention	R
Cingulo-Opercular-16_R-Ctx	R_43	Cingulo-Opercular	R
Somatomotor-15_R-Ctx	R_OP4	Somatomotor	R
Somatomotor-16_R-Ctx	R_OP1	Somatomotor	R
Auditory-02_R-Ctx	R_52	Auditory	R
Cingulo-Opercular-17_R-Ctx	R_PFcm	Cingulo-Opercular	R
Cingulo-Opercular-18_R-Ctx	R_PoI2	Cingulo-Opercular	R
Cingulo-Opercular-19_R-Ctx	R_FOP4	Cingulo-Opercular	R
Cingulo-Opercular-20_R-Ctx	R_MI	Cingulo-Opercular	R
Frontoparietal-20_R-Ctx	R_AVI	Frontoparietal	R
Orbito-Affective-02_R-Ctx	R_AAIC	Orbito-Affective	R
Cingulo-Opercular-21_R-Ctx	R_FOP1	Cingulo-Opercular	R
Cingulo-Opercular-22_R-Ctx	R_FOP3	Cingulo-Opercular	R
Somatomotor-19_R-Ctx	R_FOP2	Somatomotor	R
Dorsal-Attention-05_R-Ctx	R_PFt	Dorsal-Attention	R
Dorsal-Attention-06_R-Ctx	R_AIP	Dorsal-Attention	R
Default-23_R-Ctx	R_PreS	Default	R
Default-24_R-Ctx	R_H	Default	R
Language-06_R-Ctx	R_A5	Language	R
Language-07_R-Ctx	R_STSdp	Language	R
Default-27_R-Ctx	R_STSvp	Default	R
Frontoparietal-21_R-Ctx	R_TE1p	Frontoparietal	R
Dorsal-Attention-09_R-Ctx	R_PHT	Dorsal-Attention	R
Visual2-18_R-Ctx	R_PH	Visual2	R
Language-08_R-Ctx	R_TPOJ1	Language	R
Posterior-Multimodal-03_R-Ctx	R_TPOJ2	Posterior-Multimodal	R
Posterior-Multimodal-04_R-Ctx	R_TPOJ3	Posterior-Multimodal	R
Visual1-03_R-Ctx	R_DVT	Visual1	R
Dorsal-Attention-10_R-Ctx	R_PGp	Dorsal-Attention	R
Frontoparietal-23_R-Ctx	R_IP1	Frontoparietal	R
Dorsal-Attention-11_R-Ctx	R_IP0	Dorsal-Attention	R
Cingulo-Opercular-23_R-Ctx	R_PFop	Cingulo-Opercular	R
Cingulo-Opercular-24_R-Ctx	R_PF	Cingulo-Opercular	R
Frontoparietal-24_R-Ctx	R_PFm	Frontoparietal	R
Default-31_R-Ctx	R_PGi	Default	R
Default-32_R-Ctx	R_PGs	Default	R
Visual2-23_R-Ctx	R_FST	Visual2	R
Visual2-26_R-Ctx	R_VMV2	Visual2	R
Default-34_R-Ctx	R_31pd	Default	R
Cingulo-Opercular-25_R-Ctx	R_PoI1	Cingulo-Opercular	R
Somatomotor-20_R-Ctx	R_Ig	Somatomotor	R
Cingulo-Opercular-26_R-Ctx	R_FOP5	Cingulo-Opercular	R
Frontoparietal-27_R-Ctx	R_p47r	Frontoparietal	R
Auditory-07_R-Ctx	R_A4	Auditory	R
Frontoparietal-28_R-Ctx	R_TE1m	Frontoparietal	R

Cingulo-Opercular-28_R-Ctx	R_a32pr	Cingulo-Opercular	R
Cingulo-Opercular-29_R-Ctx	R_p24	Cingulo-Opercular	R
