# Supplementary material for: Constructing neural network models from brain data reveals representational transformations linked to adaptive behavior
Source: Nat Commun. 2022 Feb 3;13:673. doi: 10.1038/s41467-022-28323-7 (PMC8814166; doi:10.1038/s41467-022-28323-7)
Supplement: Supplementary file 2 — Reporting Summary [file 41467_2022_28323_MOESM2_ESM.pdf]

nature research

Corresponding author(s): Takuya Ito
Last updated by author(s): Dec 20, 2021

Reporting Summary

Nature Research wishes to improve the reproducibility of the work that we publish. This form provides structure for consistency and transparency in reporting. For further information on Nature Research policies, see our Editorial Policies and the Editorial Policy Checklist.

Statistics

For all statistical analyses, confirm that the following items are present in the figure legend, table legend, main text, or Methods section.

n/a	Confirmed	
☐	☒	The exact sample size (*n*) for each experimental group/condition, given as a discrete number and unit of measurement
☐	☒	A statement on whether measurements were taken from distinct samples or whether the same sample was measured repeatedly
☐	☒	The statistical test(s) used AND whether they are one- or two-sided<br>*Only common tests should be described solely by name; describe more complex techniques in the Methods section.*
☐	☒	A description of all covariates tested
☐	☒	A description of any assumptions or corrections, such as tests of normality and adjustment for multiple comparisons
☐	☒	A full description of the statistical parameters including central tendency (e.g. means) or other basic estimates (e.g. regression coefficient) AND variation (e.g. standard deviation) or associated estimates of uncertainty (e.g. confidence intervals)
☐	☒	For null hypothesis testing, the test statistic (e.g. *F*, *t*, *r*) with confidence intervals, effect sizes, degrees of freedom and *P* value noted<br>*Give P values as exact values whenever suitable.*
☒	☐	For Bayesian analysis, information on the choice of priors and Markov chain Monte Carlo settings
☒	☐	For hierarchical and complex designs, identification of the appropriate level for tests and full reporting of outcomes
☐	☒	Estimates of effect sizes (e.g. Cohen's *d*, Pearson's *r*), indicating how they were calculated

Our web collection on statistics for biologists contains articles on many of the points above.

Software and code

Policy information about availability of computer code

Data collection	Code for data collection (e.g. experimental design) was written in E-Prime 2.0.10.353. Data processing code was implemented with the HCP preprocessing pipelines.
Data analysis	Code was primarily written using scientific python packages, which are referenced in the manuscript (including version numbers). All code related to the results in the manuscript are provided on a publicly available GitHub repository: https://github.com/ito-takuya/sr_enn<br><br>We also used publicly available software packages, including PyTorch (version 1.0.1), Scikit-learn (version 0.20.3), NumPy (version 1.17.0), Scipy (version 1.5.0).

For manuscripts utilizing custom algorithms or software that are central to the research but not yet described in published literature, software must be made available to editors and reviewers. We strongly encourage code deposition in a community repository (e.g. GitHub). See the Nature Research guidelines for submitting code & software for further information.

Data

Policy information about availability of data

All manuscripts must include a data availability statement. This statement should provide the following information, where applicable:

- Accession codes, unique identifiers, or web links for publicly available datasets
- A list of figures that have associated raw data
- A description of any restrictions on data availability

As stated in the manuscript: "All data related to this study are publicly available on OpenNeuro (https://openneuro.org/datasets/ds003701)."

Field-specific reporting

Please select the one below that is the best fit for your research. If you are not sure, read the appropriate sections before making your selection.

☒ Life sciences ☐ Behavioural & social sciences ☐ Ecological, evolutionary & environmental sciences

For a reference copy of the document with all sections, see nature.com/documents/nr-reporting-summary-flat.pdf

Life sciences study design

All studies must disclose on these points even when the disclosure is negative.

Sample size	n=96 subjects. Originally, this sample size was selected to study individual differences. The sample size was chosen in order to have 80% power for individual difference analyses. The current study focuses on group-level analyses, which are better powered relative to individual-difference analyses, and is thus sufficiently powered.
Data exclusions	Technical error during MRI acquisition resulted in removing six participants from the study. Four additional participants were removed from the study because they did not complete the behavior-only session. fMRI analysis was performed on the remaining 96 participants (54 females).
Replication	Replication on an independent data set was not performed.
Randomization	Not relevant. No group differences were studied.
Blinding	Not relevant, since this was not a 1) randomized control trial; 2) no group differences were studied.

Reporting for specific materials, systems and methods

We require information from authors about some types of materials, experimental systems and methods used in many studies. Here, indicate whether each material, system or method listed is relevant to your study. If you are not sure if a list item applies to your research, read the appropriate section before selecting a response.

Materials & experimental systems

n/a	Involved in the study	
☒	☐	Antibodies
☒	☐	Eukaryotic cell lines
☒	☐	Palaeontology and archaeology
☒	☐	Animals and other organisms
☐	☒	Human research participants
☒	☐	Clinical data
☒	☐	Dual use research of concern

Methods

n/a	Involved in the study	
☒	☐	ChIP-seq
☒	☐	Flow cytometry
☐	☒	MRI-based neuroimaging

Human research participants

Policy information about studies involving human research participants

Population characteristics	Data were collected from 106 human participants across two different sessions (a behavioral and an imaging session). Participants were recruited from the Rutgers University-Newark community and neighboring communities. Technical error during MRI acquisition resulted in removing six participants from the study. Four additional participants were removed from the study because they did not complete the behavior-only session. fMRI analysis was performed on the remaining 96 participants (54 females). All participants gave informed consent according to the protocol approved by the Rutgers University Institutional Review Board. The average age of the participants that were included for analysis was 22.06, with a standard deviation of 3.84.
Recruitment	Recruitment was carried out using e-mail listserves, paper brochures submitted to different campus halls, and classroom announcements. We excluded participants that were not right-handed and were non-native English speakers. Non-native English speakers were unlikely to perform the task (or perform worse) since task instructions were provided in English. Participants were compensated at a rate of $15/hour for behavioral sessions, and $30/hour for imaging sessions.
Ethics oversight	All participants gave informed consent according to the protocol approved by the Rutgers University Institutional Review Board.

Note that full information on the approval of the study protocol must also be provided in the manuscript.

Magnetic resonance imaging

Experimental design

Design type	Includes both task-state and resting-state fMRI. We employed a mixed block/event design.
Design specifications	128 blocks, where each block contains an encoding period followed by 3 trials/events. Each subject performs 384 trials in total.<br>Blocks last for 36 TRs (sampling rate 785ms).
Behavioral performance measures	We recorded a finger button press at each trial. Subjects' mean performance across all trials performed in the scanner was 84% (median=86%) with a standard deviation of 9% (min=51%; max=96%). All subjects performed statistically above chance (25%).

Acquisition

Imaging type(s)	Functional
Field strength	3T
Sequence & imaging parameters	Data were collected at the Rutgers University Brain Imaging Center (RUBIC).<br>Whole-brain multiband echo-planar imaging (EPI) acquisitions were collected with a 32-channel head coil on a 3T Siemens Trio MRI scanner with TR=785 ms, TE=34.8 ms, flip angle=55°, Bandwidth 1924/Hz/Px, in-plane FoV read=208 mm, 72 slices, 2.0 mm isotropic voxels, with a multiband acceleration factor of 8. Whole-brain high-resolution T1-weighted and T2-weighted anatomical scans were also collected with 0.8 mm isotropic voxels. Spin echo field maps were collected in both the anterior to posterior direction and the posterior to anterior direction in accordance with the Human Connectome Project preprocessing pipeline. A resting-state scan was collected for 14 minutes (1070 TRs), prior to the task scans. Eight task scans were subsequently collected, each spanning 7 minutes and 36 seconds (581 TRs). Each of the eight task runs (in addition to all other MRI data) were collected consecutively with short breaks in between (subjects did not leave the scanner).
Area of acquisition	Whole brain

Diffusion MRI ☐ Used ☒ Not used

Preprocessing

Preprocessing software	Resting-state and task-state fMRI data were minimally preprocessed using the publicly available Human Connectome Project minimal preprocessing pipeline version 3.5.0.
Normalization	[Nonlinear] spatial normalization to standard template and intensity normalization (in accordance with the HCP preprocessing pipelines)
Normalization template	MNI152
Noise and artifact removal	After minimal preprocessing, additional custom preprocessing was conducted on CIFTI 64k grayordinate standard space for vertex-wise analyses using a surface based atlas. This included removal of the first five frames of each run, de-meaning and de-trending the time series, and performing nuisance regression on the minimally preprocessed data. We removed motion parameters and physiological noise during nuisance regression. This included six motion parameters, their derivatives, and the quadratics of those parameters (24 motion regressors in total). We applied aCompCor on the physiological time series extracted from the white matter and ventricle voxels (5 components each extracted volumetrically). We additionally included the derivatives of each component time series, and the quadratics of the original and derivative time series (40 physiological noise regressors in total). This combination of motion and physiological noise regressors totaled 64 nuisance parameters, and is a variant of previously benchmarked nuisance regression models.
Volume censoring	We did not perform volume censoring.

Statistical modeling & inference

Model type and settings	We used multiple statistical models/inference techniques. They are extensively detailed in the following Methods sections:<br>fMRI decoding: Identifying sensory stimulus representations<br>fMRI decoding: Identifying task rule representations<br>fMRI activation analysis: Identifying motor response activations<br>fMRI representational similarity analysis: Identifying conjunction hubs<br>Statistical and permutation testing of predicted motor response activations
Effect(s) tested	The effects tested were primarily cross-validated decoding accuracies

Specify type of analysis: ☒ Whole brain ☐ ROI-based ☐ Both

Statistic type for inference (See Eklund et al. 2016)	Non-parametric (permutation-based) testing of decoding accuracies. P-values were further assessed using multiple comparisons via FDR correction
Correction	FDR

Models & analysis

n/a	Involved in the study
☐	☒ Functional and/or effective connectivity
☒	☐ Graph analysis
☐	☒ Multivariate modeling or predictive analysis

Functional and/or effective connectivity	Multiple linear regression
Multivariate modeling and predictive analysis	Independent variables: Task conditions and behavioral responses (finger presses)<br>Dependent variables: fMRI activations
